# An Update on Breast Cancer Multigene Prognostic Tests—Emergent Clinical Biomarkers

**DOI:** 10.3389/fmed.2018.00248

**Published:** 2018-09-04

**Authors:** André Filipe Vieira, Fernando Schmitt

**Affiliations:** ^1^IPATIMUP - Epithelial Interactions in Cancer Group, Instituto de Patologia e Imunologia Molecular, Universidade do Porto, Porto, Portugal; ^2^Instituto de Investigação e Inovação em Saúde, Universidade do Porto, Porto, Portugal; ^3^Faculdade de Medicina, Universidade do Porto, Porto, Portugal

**Keywords:** molecular signatures, genetic assays, prognostic tests, breast cancer, biomarkers

## Abstract

Multigene signatures generate crucial prognostic information particularly useful for cancer patients where clinical parameters and traditional immunohistochemical markers alone lead to equivocal prognosis. Clinicians are now provided with molecular tools that assist in the outline of adjuvant therapies, namely helping decide on the extension of adjuvant endocrine therapy or on suppressing adjuvant chemotherapy in patients were toxic effects are particularly deleterious or when this treatment is fundamentally not needed. The importance of cancer multigene prognostic signatures is well elucidated in the guidelines for adjuvant systemic therapy in early-stage breast cancer and the guidelines on disease staging that are progressively integrating gene expression assays as classification biomarkers. In addition to the predictive and prognostic value, some genetic tests provide intrinsic subtyping classification. Herewith, we compare the molecular tests OncotypeDX, MammaPrint, Prosigna, EndoPredict, Breast Cancer Index, Mammostrat, and IHC4 and report the eligibility of each one in the suitable setting. Through to now, there is not a commercially available multigene test that makes recommendations regarding adjuvant treatment for HER-2 and triple negative breast cancers. Thus, these patients still receive adjuvant chemotherapy. Importantly, triple negative carcinomas are very heterogeneous regarding prognosis and new molecular signatures that decipher this very heterogeneous subgroup of breast cancer may improve the clinical management of the disease.

## Introduction

The clinical course of breast cancer may be difficult to predict as this malignancy is composed of many biological subtypes that in turn exhibit intratumor heterogeneity, and patients often present at different stages of pathological development. In spite of this, a limited number of prognostic factors have a crucial role today to assess potential recurrence or death from breast cancer. Patient age, tumor size, comorbidity, tumor grade, and number of metastasized axillary lymph nodes are the strongest prognostic factors. One validated algorithm-based model to estimate overall survival (OS) and 10-year disease-free survival (DFS) that incorporates most of the aforementioned prognostic factors is Adjuvant! Online (www.adjuvantonline.com) (1, 2). Clinicians are occasionally faced by high-risk breast cancer patients in an early stage of disease, with estrogen-receptor (ER)-positive breast cancer, without axillary lymph node involvement, or involvement of up to 3 lymph nodes. The decision to administer adjuvant chemotherapy or extended adjuvant endocrine therapy to these patients is equivocal. Thus, biomarkers to improve the clinical benefit of adjuvant therapies in patients with late recurrence are clinically valuable. Today, breast cancer management is already changing in light of the new molecular analysis that is becoming more accessible in day-to-day pathology labs. Genetic prognostic tests are biomarkers commercially available in the form of medical devices/tests. The prognosis of breast cancer disease, the assessment of patients where chemotherapy will be beneficial, as well as the identification of the molecular subtype can be provided by such molecular tests.

This review focuses on seven major prognostic signatures for breast cancer (OncotypeDX, Mammaprint, Prosigna, EndoPredict, Breast Cancer Index, Mammostrat and IHC4) validated through clinical trials, some of which are already approved by FDA and recommended by American [National Comprehensive Cancer Network (NCCN), American Society of Clinical Oncology (ASCO)] and European [European Society of Medical Oncology (ESMO)] guidelines committees. Further, we explore the future in the development of novel molecular prognostic tests, namely in subgroups of mixed behavior breast cancers, such as the triple-negative carcinomas.

## Breast cancer molecular subtypes

DNA microarrays and Next Generation Sequencing (NGS) represent a great advance in molecular technology. The characterization of breast cancer by DNA microarray analysis has revealed crucial classification systems by gene expression profile (3). Five major subtypes of breast carcinomas were identified: ER-positive/HER2-negative (luminal A and luminal B subtypes); ER-negative/HER2-negative (basal subtype); HER2-positive; and carcinomas that have features similar to normal breast tissue (4–6). Differing relapse-free survival (RFS) and OS have been found for these intrinsic molecular subtypes in several retrospective studies. Further, other breast carcinomas have been identified as a molecularly distinct disease, as is the case of claudin-low cancers (7), metaplastic (8), molecular apocrine (9), and invasive lobular carcinomas (10). Triple negative carcinomas, which are ERα-negative, PgR-negative and HER2 negative by immunohistochemistry, have been shown to be heterogeneous regarding response to treatment (11) and were recently subdivided into molecular subtypes (12).

## Devising multigene prognostic signatures from gene expression analysis

Big data provided by DNA microarray technologies and RNA sequencing of breast carcinomas in combination with bioinformatics provide unparalleled opportunities for studying breast carcinomas. In the past decade, algorithms have been generated estimating the rates of cancer recurrence and/or survival that comprise a reduced set of genes that constitutes the gene signature. The genetic signature is obtained by computer-based models, validated in clinical studies and, in some cases, translated to commercial prognostic assays (13).

To develop a molecular gene signature, gene expression variations are determined within the candidate expression dataset. High variance genes or genes that are found differentially expressed between selected phenotypically diverse groups (e g., good prognosis vs. bad prognosis) are selected. Patients are grouped into the categories based on sorted gene expression profiles, usually translated into a score. The Kaplan-Meier estimator, logistic regression, and Cox proportional hazards model are well-established statistical approaches to test the survival functions that measure distant recurrence-free survival (DRFS), DFS or OS during time in large-scale data sets of gene expression with clinical data, such as survival or therapeutic response. When assembling a predictive signature, the expression values of the genes present in the signature are weighted to improve its predictive success. A mathematical equation is built that predicts 5-year or 10-year post diagnosis risk of recurrence/death. These strategies allow to find a small subset of gene alterations that are most informative for survival prediction (14).

In the Kaplan Meier method, an estimation of the survival function during time is analyzed by log-rank statistics and the fraction of patients surviving at each time after surgery and/or treatment is plotted (15, 16). In logistic regression, a statistical regression method is applied where the independent variable determines an outcome in which there are only two possible outcomes (alive or deceased; relapse, or no relapse). It was demonstrated that the predictive accuracy could be markedly enhanced by the application of logistic regression analysis, in comparison to conventional Kaplan Meier approaches, which are often based upon incomplete or greatly right-censored clinical data (17, 18). The Cox proportional hazards model is a statistical regression model that allows investigating the effect of several variables that may impact patient outcome and it assumes a constant risk of death/relapse during time, known as the hazard/odds ratio (19), in contrast to the Kaplan Meier estimator that assesses the impact of a single factor in a varying proportion of deceased/relapsed patients during time (16).

The gene signature has to be validated in clinical assays, where a risk score can stratify patients, according with the probability of survival given by the survival functions. In general, patients are assigned to a low-risk group when the risk of recurrence is about 10% (10-year survival probability around 90%) (13). The gene signature can be tested for the interaction between the treatment benefit (e.g., chemotherapy) and the risk score in Cox proportional hazards and/or Kaplan-Meier models. In addition to the assessment of the treatment effect in survival, models can be created adding the risk score to clinical variables, such as tumor size, age, and grade. The performance of survival mathematical models can greatly improve with the combination of both the gene signature plus clinical and pathological data.

Some molecular signatures have been translated to a short list of protein biomarkers that can be readily tested through immunohistochemistry of immunofluorescence (20, 21). This is useful in pathology laboratories where routine molecular techniques are being slowly implemented. An algorithm is calculated based on the survival regression models to assess the coefficients and the establishment of a prognostic score/index in tissues.

The implementation of high-throughput techniques like free circulating DNA profiling and micro-RNA analysis will permit the development of multivariate models and open avenues for new gene expression signatures to be formulated and implemented.

## The clinical application of genetic tests: prognostic information, therapy decision, molecular subtyping, and patient staging

Ideally, genetic tests must be able to accurately measure the gene profile of interest in different certified laboratories. This requisite is known as analytic validity of the assay and it must be maintained as prognostic tests become decentralized, i.e., adapted in house to each laboratory, preferably using formalin–fixed paraffin embedded (FFPE) tissue samples. Genetic tests must also provide clinical validity, as they should be able to clearly stratify a population into two or more groups of patients that have different clinical behavior regarding patient outcome—usually RFS, DRFS, or OS. Finally, the clinical utility of genetic tests is shown in appropriately designed clinical trials that dictate whether using genetic tests leads to optimized clinical decision-making with a confident degree of evidence. Genetic tests patient prognostic assessment should be demonstrated in retrospective or prospective studies. Furthermore, genetic tests should be assessed for predictive value through the evaluation of treatment benefit, ideally in prospective studies (13).

**Figure 1:**
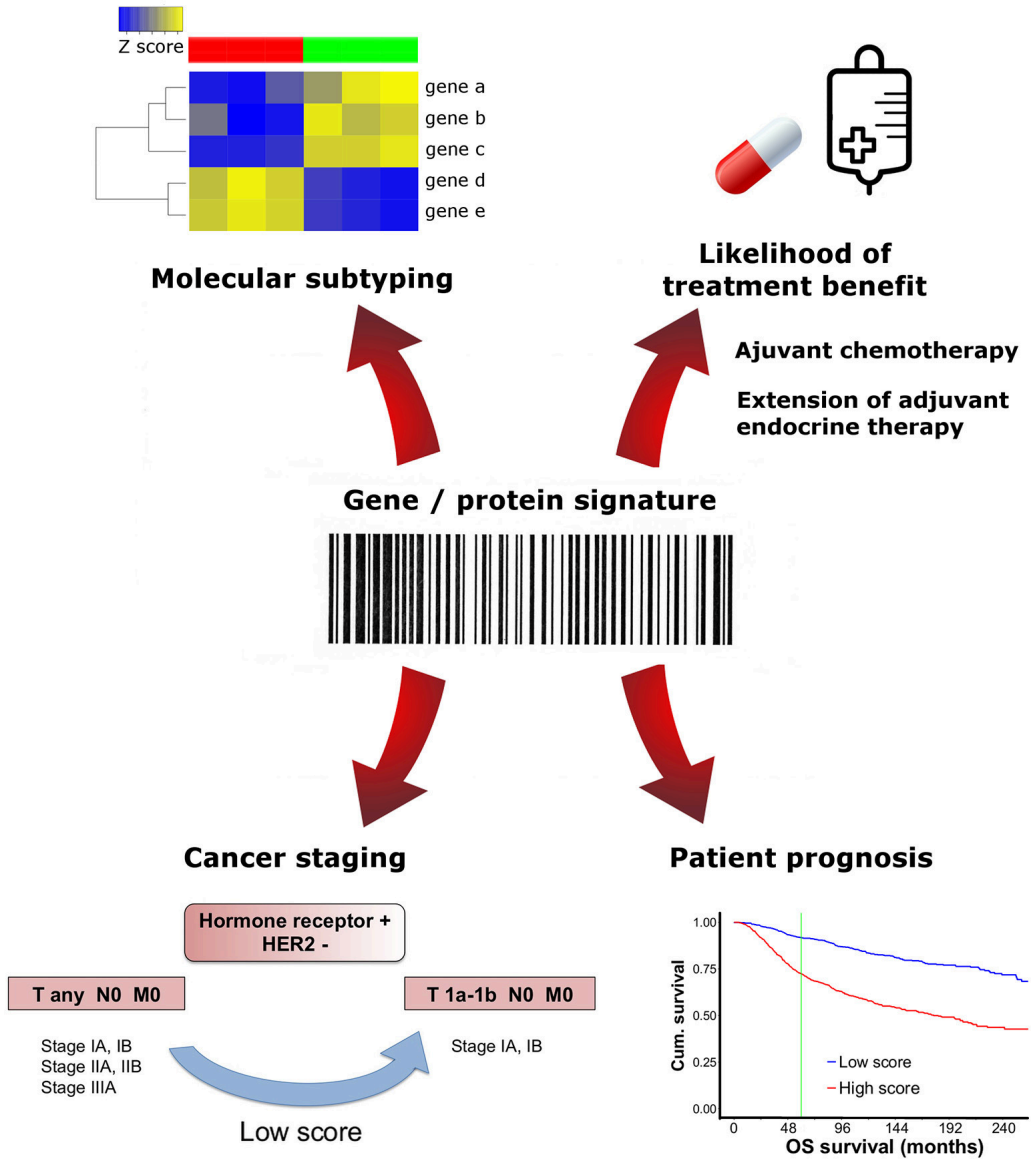
Clinical applications of multigene/protein signatures in breast cancer. Different multigene/protein assays may have distinctive applications. Molecular signatures can be used to test prognosis, predict treatment benefit, determine tumor subtype or downstage select patients.

Today, the applications of gene expression signatures in the clinic are diverse and these assays have the propensity to assume a prominent or even critical significance in every pathology laboratory (Figure 1).

In 2017, AJCC recognized the need to incorporate gene expression prognostic panels into the TNM staging system (eighth edition) (22). Although the expert panel does not endorse any particular assay, it is clear that genomic assay recurrence scores can alter prognosis and stage. Low risk scores given by OncotypeDX, Mammaprint, Endopredict, PAM50/Prosigna, or Breast Cancer Index (BCI) can be used regardless of the tumor size, to downstage hormone receptor-positive, HER2 negative and lymph node-negative tumors, placing them into the same prognostic category as T1a-T1b N0 M0 carcinomas. As of this time, no upstaging is recommended based on multigene panel testing (22).

In 2016, ASCO guidelines (additional update in 2017) made several recommendations regarding the decision of suppressing adjuvant systemic therapy for women with early-stage invasive luminal breast cancer (23, 24). Importantly, this therapy has a crucial impact in reducing tumor growth and patient cancer mortality, thus clinicians should be cautious when deciding to opt out of this therapy. In addition to the clinician, the patient is also involved in health care decision-making and the latter should be informed of the risks and benefits of any treatment modification. Still, for some women the toxicity associated with adjuvant chemotherapy may not justify the clinical benefits obtained with such therapy. None of the gene expression or protein assays are recommended by ASCO, NCCN, or ESMO regarding decision-making on HER2-positive breast cancer or triple negative breast cancers (23–27).

Another potential application of multiparameter gene expression assays is the decision to extend endocrine therapy in patients with ER/PgR-positive, HER2-negative, node-negative breast cancer and with 5 years of endocrine therapy without disease recurrence. Noteworthy, ASCO does not make any recommendation as to a specific assay to make decisions on extended endocrine therapy (23).

Furthermore, certain gene signatures were devised to identify breast cancer molecular subtypes, which may help in accessing prognosis. PAM50/Prosigna (28) and BluePrint (29) are an example of such tests. Of note, multigene tests and molecular subtype classification do not inform us about the mutations and epigenetic events that have impact in cancer progression. Some authors argue in favor of assays that are based in the combination of mutation profiling with the gene expression analysis (30) because the presence of specific driver genetic aberrations can predict the response to specific targeted therapies (30, 31). Thus, one important approach is to assess the complete spectrum of cancer mutations and find the specific actionable molecules that are crucial in order to perform tailored therapy. Today, two companies commercialize molecular tests that evaluate distinct gene mutational profiles and predict actionable targets for the clinic. These tests are not focused in the identification of a simple gene or protein signature. Specifically, Foundation One CDx (Foundation Medicine, Cambridge, Massachussets, US and Roche, Basel, Switzerland) is a FDA approved NGS-based *in vitro* diagnostic assay that gives an informative comprehensive genomic profile of the patient's tumor, analyzing all classes of gene mutations known to be somatically altered in solid carcinomas and allowing the matching with targeted therapies. It detects base substitutions, insertion and deletion events (indels), copy number alterations and select gene rearrangements in 324 genes, as well as genomic phenotypes including microsatellite instability and tumor mutational burden, using DNA isolated from FFPE tumor tissue specimens. Another test providing precision medicine is Caris Molecular Intelligence (Caris Life Sciences, Phenix, Arizona, US) that uses multiple tumor profiling technologies to retrieve information from patient's DNA (base substitutions, indels and copy number alterations), RNA (gene fusions and variant transcripts) and protein (immunohistochemistry). Like the aforementioned assay, Caris Molecular Intelligence tumor profiling includes tumor mutational burden and microsatellite instability testing via NGS (32).

Up to now, there is no molecular test that predicts the site of distant metastasis formation. For example, luminal breast tumors frequently disseminate to the bone, whereas, the brain is an organ preferentially targeted by HER2 and basal-like tumors cells. A gene signature that predicts the site of relapse could lead to closer vigilance of potentially implicated organs.

## Molecular signatures and their validation in evidence-based clinical trials

ASCO and NCCN make specific recommendations based on clinical studies and the level of evidence they provide on the use of genetic assays to help clinicians decide on adjuvant therapy for women with early stage invasive breast cancer (Table 1).

**Table 1:** Clinical trials implicated in the development of multigene prognostic signatures.

	**Number of genes/proteins**	**Candidate patients for adjuvant chemotherapy assessment (recommended by guidelines)**	**ASCO / NCCN recommendations**	**AJCC staging**	**Molecular subtyping**	**Clinical trial**	**Combination with clinical parameters in score assessement**	**References**


OncotypeDX	21 genes (16 genes + 5 reference genes)	ER/PgR+, HER2, node – ER/PgR+, HER2, node +	Yes (strong)	Yes	No	NSABP B14 (retropective) NSABP B20 (retrospective) SWOG 8814 (retrospective) TransATAC (retrospective) TAILORx (prospective) RxPonder (prospective)	No	(33) (34) (35) (36) (37, 38) (39)
MammaPrint	70 genes	ER/PgR+, HER2, node – ER/PgR+, HER2, node +	Yes (strong)	Yes	Yes (BluePrint)	TRANSBIG (retrospective) RASTER (prospective) MINDACT (prospective)	Yes (Adjuvant! Online)	(40) (41) (42)
Prosigna/PAM50	50 genes (+ 5 reference genes)	ER/PgR+, HER2-, node -	Yes (moderate)	Yes	Yes	ABCG8 (retrospective) ATAC (retrospective)	Yes (Proliferation score, tumor size)	(43) (36)
EndoPredict	12 genes (8 genes + 3 RNA reference genes + 1 DNA reference gene)	ER/PgR+, HER2-, node -	Yes (moderate)	Yes	No	GEICAM 9906 ABCSG6 (retrospective) ABCSG8 (restrospective)	Yes (tumor size and nodal status (EPclin))	(44) (45) (46)
Breast Cancer Index	7 genes (5 genes + 2 genes ratio)	ER/PgR+, HER2-, node -	Yes (moderate)	Yes	No	TransATAC (retrospective) Stockholm trial (retrospective)	No	(47) (48)
Mammostrat	5 proteins	–	No	No	No	NSABP B14 (retrospective) B20 (retrospective)	No	(49)
IHC4	4 proteins	–	No	No	No	TransATAC (retrospective)	Yes (nodal status, tumor size, grade, and age)	(21) (50)

*Molecular assays can be used to determine the benefits of early stage breast cancer chemotherapy (ASCO and NCCN guidelines) and to optimize the staging of patients (AJCC TNM staging 8th edition guidelines)*.

### Oncotype DX

Oncotype DX (Genomic Health, Redwood, CA) is a 21-gene signature that is one of the best-validated breast cancer multigene tests. It is incorporated in the staging guidelines of AJCC 8th edition (22), as well as in ASCO therapy guidelines for early stage breast cancer treatment (23, 24), NCCN clinical practice guidelines in oncology (26), ESMO clinical practice guidelines for diagnosis, treatment, and follow-up of primary breast cancer (25) and St. Gallen consensus panel guidelines (51). Oncotype DX is based on RNA isolation from FFPE breast cancer tissue followed by RT-PCR, providing a stratification of the 5-year or 10-year risk of distant relapse into risk groups: low risk where the clinical benefit of chemotherapy is expected to be small [recurrence score (RS < 18)], intermediate risk where it is uncertain whether the beneficial effect of chemotherapy outbalance the risks and complications mediated by its toxic lateral effects (RS 18–31), and high risk where there is a high probability of cancer of recurrence, and the benefits of chemotherapy are should surpass the risks of side effects (RS >31). Of note, in the latest clinical trials risk scores cutoffs have been optimized, reflecting a forthcoming adjustment in the assay (37).

The assay is FDA cleared and it was initially tested in node negative patients using samples from the NSABP B14 clinical trial (33). The assessment of chemotherapy benefit was done in NSABP B20 study (34) and in the larger studies SWOG 8814 (35) and TransATAC (36).

In the retrospective analysis performed on the SWOG 8814 study, a randomized clinical trial in post-menopausal, axillary lymph node-positive, ER-positive breast cancer women, Oncotype DX delivered predictive evidence for chemotherapy benefit in tamoxifen treated patients (35).

The TAILORx study is a prospective phase III trial designed for HR-positive, HER-2 negative and node negative breast cancer (38, 52). The RS boundaries that were initially determined for Oncotype DX were modified in this study to avoid undertreatment, with the lower limit going from 18 to 11 and the upper end was redefined from 31 to 25. The initial results from TAILORx showed that women with HR positive, HER2 negative, and node-negative breast cancer in the low RS group have a very low risk of recurrence at 5 years (< 10%) with endocrine therapy alone, and therefore, can safely omit chemotherapy (37). Recently, Sparano and colleagues reported the definitive results from TAILORx, clarifying the effect of chemotherapy for women considered to be at intermediate risk for recurrence. Patients in this group were randomized to receive endocrine therapy with or without chemotherapy. The authors established that chemotherapy may be spared in all women older than 50 with RS results of 11 to 25 and all women age 50 or younger with RS results of 11–15 (52).

In reference to the 21-gene signature, ASCO guidelines report that “chemotherapy is indicated in early stage patients that have ER/PgR–positive, HER2-negative, node-negative breast cancer with a high RS and it is not indicated in patients with a low RS.” In patients with an intermediate RS, the assessment is not direct and recommendations may be determined by TAILORx. In these cases, the likelihood of distant recurrence and benefit from chemotherapy increases with an increase in the RS result. For ER/PgR–positive node-positive breast cancer, ASCO guidelines are cautious about using Oncotype DX assay. Additional studies are required to identify patients with different extent of axillary nodal status and RS where chemotherapy is in fact beneficial (23). An ongoing trial (RxPONDER, ClinicalTrials.gov identifier: NCT01272037) is trying to identify the cutoff of the RS for which adjuvant chemotherapy is advantageous for axillary lymph node positive patients. According with NCCN guidelines, “the 21-gene RT-PCR assay can be considered in patients with 1–3 involved ipsilateral axillary lymph nodes to guide the addition of combination chemotherapy to standard hormone therapy” (26). A retrospective analysis of prospective randomized trials (NSABP B14 and B20, SWOG 8814 and TransATAC) suggests that Oncotype DX as a similar prediction ability in these patients as in the patients lacking lymph node involvement (26). Both recommendation guidelines indicate that the 21-gene RS should not be used to guide treatment decision in HER2-positive breast cancer or triple-negative breast cancer (23, 24, 26).

### Mammaprint

MammaPrint is a 70-gene signature endorsed in the staging guidelines of AJCC 8th edition (22), as well as in ASCO guidelines for early stage breast cancer treatment (23, 24), NCCN clinical practice guidelines in oncology (26), ESMO clinical practice guidelines for diagnosis, treatment, and follow-up of primary breast cancer (25) and St. Gallen consensus panel guidelines (51). MammaPrint is a prognostic test cleared by the FDA to stratify patients with ER-positive or ER-negative breast carcinomas into a high vs. low risk for relapse (53).

In the TRANSBIG consortium study, the Mammaprint gene score proved to be better at stratifying low risk vs. high risk patients than the clinical risk assessed with the Adjuvant! Online tool (40). Prospective validation in node negative patients was obtained in the RASTER trial, where clinical high risk patients but with a low MammaPrint genetic risk without chemotherapy did not negatively impact in DMFS (41). Recently, prospective indication of the predictive ability of MammaPrint in early-stage luminal breast cancer for adjuvant chemotherapy became available in the MINDACT trial (level1A evidence). The MINDACT study included 6,693 women with early stage breast cancer (lymph node negative or 1-3 lymph node positive). This study showed that chemotherapy could be spared in women who had a low genomic risk for recurrence according to MammaPrint and who were at high clinical risk for relapse defined using Adjuvant! (42). In a subset of same clinical trial, a study presented at ESMO 2017 showed that the 70-gene signature MammaPrint could detect aggressive small tumors [tumor size < 1 cm (pT1abpN0)]. The authors found that around 25% of small tumors were aggressive and patients benefited from chemotherapy (54). The MINDACT study further provided the platform for MammaPrint to be included in ASCO guidelines for clinicians to direct chemotherapy in node positive early stage breast cancer patients (23, 24). According with the recently updated ASCO guidelines for breast cancer treatment, if a patient has HR–positive, HER2-negative, bearing a node negative carcinoma, but with high clinical risk, MammaPrint can be used to guide therapy decisions. ASCO states that “MammaPrint assay may also be used in patients with one to three positive nodes and a high clinical risk to inform decisions on withholding adjuvant systemic chemotherapy. However, such patients should be informed that a benefit from chemotherapy cannot be excluded.” Further, “if a patient has triple negative breast cancer, the clinician should not use the MammaPrint assay to guide decisions on adjuvant systemic chemotherapy” (24). Of note, the MammaPrint index is positively associated with the likelihood of PCR, i e., high index patients benefit from neoadjuvant chemotherapy (29). Interestingly, MammaPrint could provide prognostic value in HER2-positive breast cancer. However, the 10-year distant DFS is 84%, a value that is not favorable to suppress adjuvant chemotherapy. Thus, the use of the MammaPrint prognostic test to decide the administration of adjuvant chemotherapy in HER2-positive breast cancer patients is not recommended and additional studies are required (55).

Likewise to ASCO guidelines, the 2017 St. Gallen International Breast Cancer panel (ESMO) expanded its guidelines to recommend the use of MammaPrint to help guide chemotherapy decision-making for patients with early-stage breast cancer, with HR-positive and lymph-node positive breast cancer. Oncotype DX gene-expression test was also recommended for guiding treatment decisions in these patients (27). Regarding the recent NCCN guidelines, the latest results from the MINDACT study have not been included. Nonetheless, NCCN states that prognostic multigene assays are to be considered to estimate risk of recurrence or death and benefits of adjuvant chemotherapy in these patients (26).

BluePrint is a molecular classification system based on 80 genes that allows breast cancer subtyping classification into low-risk luminal-type, high-risk luminal-type, HER-2-type and basal-like-type. It enables patient selection for either chemotherapy or endocrine treatment (29). Also, there is a good association between BluePrint subtyping and chemosensitivity PCR, with basal-like and HER2 subtypes having a higher PCR rates (29, 56). BluePrint differs from the PAM50 classifier as only 9 genes are present in both gene sets: *ESR1, PGR, ERBB2, GRB7, BCL2, NAT1, FOXA1, FOXC1, MLPH* but the classification of patients into luminal, HER2, and basal subgroups by PAM50 or BluePrint is expected to have great similarity, since the agreement with the original intrinsic gene set from Perou and colleagues is >90%. Of note, today there is no standardized method for molecular subtyping of breast cancer, hence, it is uncertain which methodology is ideal at classifying breast cancer molecular subtypes (29).

### Prosigna/Pam50

Prosigna/PAM50 (Prosigna Breast Cancer Prognostic Gene Signature Assay; NanoString Technologies, Seattle, WA) is a 50 genes molecular signature that was developed in premenopausal and postmenopausal women treated without any adjuvant systemic therapy (36, 43). It encompasses the NanoString nCounter technology in patient analysis. This test provides a risk of recurrence score (ROR) that takes into account the PAM50 profile described by Parker et al. (28) and clinical features of the patient, such as tumor size and proliferation score (33). ROR is stratified into low (10-year distant recurrence < 10%), intermediate (10-year distant recurrence 10–20%) and high scores (10-year distant recurrence >20%). Analogously to Mammaprint/BluePrint, Prosigna/PAM50 provides breast cancer intrinsic subtype classification.

ASCO guidelines indicate that the clinician may use this signature “in conjunction with other clinicopathologic variables to guide decisions on adjuvant systemic therapy in ER/PgR-positive, HER2-negative, node-negative breast cancer: chemotherapy should be considered for patients in the PAM50 high-risk group and it is not indicated for patients in the low-risk group.” Additional studies are needed to support recommendations about adjuvant chemotherapy in patients with an intermediate Prosigna/PAM50 ROR score. Regarding node positive ER/PgR-positive, HER2-negative breast cancer, ASCO warns that “more data are required to determine whether PAM50-ROR can be used with confidence in guiding the use of adjuvant systemic therapy.” Further, no data support the use of PAM50-ROR in HER2-positive breast cancer or in triple negative breast cancer (23).

The ABCG8 study and the ATAC trials provided evidence of the prognostic use of this molecular test, with subsets comprising the retrospective analysis of the PAM50 signature in endocrine-treated patients with ER-positive, node-negative disease (36, 43). The Prosigna/PAM50 ROR score added statistically significant prognostic information beyond the standard clinical treatment score, which was derived from standard clinical covariates, including age, grade, tumor size, nodal status, and adjuvant therapy (43). In the study by Dowsett and colleagues that compared Prosigna/PAM50 with Oncotype DX in the same FFPE samples of endocrine-treated patients with ER-positive, node-negative disease, the authors showed that more information was added by Prosigna/PAM50 ROR than by Oncotype DX RS, i.e., more patients were scored as high risk and fewer as intermediate risk by ROR than by RS (50). Although the prognostic value of Prosigna/PAM50 has been clarified, there is a lack of prospective clinical studies that show the predictive value of this signature.

### Endopredict

EndoPredict (Myriad Genetics, Inc) is a twelve gene molecular signature. It comprises the measurement of the expression of eight cancer related genes, three RNA reference genes and one DNA reference gene. Endopredict calculates a risk score (EP, endopredict score), which can be used together with tumor size and nodal status to allow the calculation of a comprehensive risk score (EPclin) (45). Its applications include guiding treatment decisions for chemotherapy as well as extended anti-hormonal therapy.

Clinical evidence for the use of this signature came from the GEICAM trial that showed that EP is an independent prognostic parameter in node-positive, ER+/HER2– breast cancer patients treated with adjuvant chemotherapy followed by hormone therapy (44). EP and EPclin were also endorsed in two randomized phase III trials (ABCSG6 and ABCSG8) that comprised more than 1700 postmenopausal breast cancer patients treated with endocrine therapy alone, indicating that both EP and EPclin could be employed to stratify subgroups displaying notable differences in 10-year distant recurrence survival in patients with node-negative and node-positive disease (45).

Although the strength of recommendation is lower than that with Oncotype DX or MammaPrint studies, ASCO guidelines indicate that EP score may also be employed in the decision-making process regarding the administration of adjuvant systemic chemotherapy in patients with ER/PgR–positive, HER2-negative, node-negative breast cancer. For node positive patients, the level of evidence of the present EndoPredict studies is insufficient for ASCO to make a strong recommendation (23). Further, numbers are not available backing the use of EP or EPclin in HER2-positive breast cancer or triple negative breast cancer.

### Breast cancer index

Breast Cancer Index (BCI, Biotheranostics, Inc.) combines the expression of 5 proliferation genes known as molecular grade index (MGI) with the 2-gene ratio *HOXB13*:*IL17BR* (H:I) in a linear model. This score was developed in postmenopausal patients with ER-positive, lymph node–negative breast cancer as a predictive test for the likelihood of benefit from extended adjuvant endocrine therapy (57).

The TransATAC and the Stockholm trials provided the clinical validation and the indication of prognostic utility for this molecular signature (47, 48, 58). Retrospective analysis of tumor samples from these randomized trials allowed the BCI assay (H:I+MGI) to independently identify patients on 5- or 10 year endocrine therapy with risk of late-distant recurrence (58). Further, although studies demonstrate that BCI has clinical use regarding decision making about the extension of adjuvant endocrine therapy beyond five years in patients with ER/PgR-positive, HER2-negative, node-negative breast cancer (58), the application of this test is not recommended by ASCO guidelines, due to insufficient evidence. Further, data are not available to support the use of BCI in luminal node positive breast cancer, in HER2-positive or in triple negative breast cancer to guide decisions on adjuvant systemic therapy (23).

### Mammostrat

Mammostrat test (Clarient Diagnostic Services, a GE Healthcare company, CA) is a five-protein based assay that provides a score for low-risk, moderate-risk, and high-risk patients. Mammostrat is not a true genetic test. Rather, it is a five-antibody immunohistochemistry prognostic test. This test uses the markers CEACAM5, HTF9C, NDRG1, SLS7A5, and TP53 to group patients on tamoxifen therapy into risk groups to inform about prognosis and putative treatment choices, namely regarding the likelihood of benefit of adjuvant systemic therapy (20). Mammostrat was developed for ER/PgR-positive, early stage invasive breast cancer patients. Studies showing the support the use of the five-protein assay in HER2-positive breast cancer or TN breast cancer are lacking.

There is evidence based studies showing that Mammostrat has prognostic value in tamoxifen-treated patients, being able to recognize those patients who have superior benefit from adjuvant chemotherapy (20, 59). Still, the proportion of patients who were recurrence free at 10 years was only 85% in the low-risk subgroup (59). Thus, ASCO does not make a strong recommendation for the assessment of adjuvant chemotherapy benefit in early stage patients with luminal breast cancer (node positive or node negative) (23).

### IHC4

IHC4 is an index derived from evaluation of ER, PgR, HER2, and Ki67 by immunohistochemistry, which are translated using an algorithm into a disease recurrence risk. These four markers are already broadly used in the clinical setting to define surrogate molecular subtypes. The clinical trial TransATAC was retrospectively evaluated for the four protein markers (21). Since the validation and testing of IHC4 is limited to very few studies and it has not been shown to be sufficiently reproducible, ASCO does not recommend its general clinical application. IHC4 is not recommended to be used in triple negative or HER2 breast carcinomas (21, 23).

## Comparison of multigene prognostic tests

Despite prognostic tests having different sets of genes/proteins, some of them are shared between different signatures (Table 2 and Supplementary Table 1). Oncotype DX shares the highest amount of genes/proteins with other signatures, 9 genes being common with Prosigna/PAM50 (*BIRC5, CCNB1, MYBL2, MMP11, GRB7, ESR1, PGR, BCL, BAG1*), 1 gene with EndoPredict (*BIRC5*), and 4 genes/proteins with IHC4 (*ESR1, PGR, HER2, Ki67*). MammaPrint signature (60) shares one gene with Oncotype DX (*SCUBE2*), three genes with Prosigna/PAM50 (*KNTC2, MELK, ORC6L*) and one gene with BCI (*CENPA*). Prosigna/PAM50 signature has two genes that are present in EndoPredict signature (*BIRC5, UBE2C*), one gene that is present in BCI (*RRM2*), and two genes/proteins that are present in IHC4 (*ESR1, PGR*). Mammostrat does not have any genes/protein in common with the other signatures. In light of the above, the number of shared genes/proteins between molecular signatures is very small, but they are able to stratify patient DFS nonetheless. In fact, although the gene sets being tested in the different signatures is different, some biological functions and molecular pathways are shared. Interestingly, proliferation genes and hormone receptor related genes are repeatedly found in different breast cancer prognostic signatures. In fact, it has been suggested that most of the survival readout obtained from breast cancer signatures is derived from a proliferation phenotype (61). Of note, despite that current genetic tests have been optimized to provide the best prognostic information, work by Venet et al. suggested that a signature made from a random selection of genes from the genome has a high chance to be significantly associated with outcome, more so when the random signatures are made of more than 100 genes (61).

**Table 2:** Shared genes/proteins between molecular signatures.

	**Oncotype DX 21 genes**	**Mammaprint 70 genes**	**Prosigna/PAM50 50 genes**	**Endopredict 12 genes**	**BCI 7 genes**	**Mammostrat 5 proteins**	**IHC4 4 proteins**
Oncotype DX		SCUBE2	BIRC5, CCNB1, MYBL2, MMP11, GRB7, ESR1, PGR, BCL, BAG1	BIRC5			ESR1, PGR, HER2, Ki67
MammaPrint	–	–	KNTC2, MELK, ORC6L		CENPA		
Prosigna/PAM50	–	–		BIRC5, UBE2C	RRM2		ESR1, PGR
EndoPredict	–	–	–	–	–	–	–
BCI	–	–	–	–	–	–	–
Mammostrat	–	–	–	–	–	–	–
IHC4	–	–	–	–	–	–	–

*For the complete gene/protein list, see Supplementary Table 1*.

The fact that molecular prognostic tests are not alike means that two tests performed on the same sample may give distinct results. Further, tests that have an “intermediate” risk score lead to an inconclusive result. When applying more than one genetic test on the same sample, we have to clarify which assay is better to guide treatment decisions. In a study by Fan and colleagues the concordance of MammaPrint and Oncotype DX assays in terms of patients assigned to the same risk category was 77% for those with ER-positive disease (62). Data presented in Miami Breast conference in 2014 showed that the concordance between MammaPrint risk groups and Oncotype DX categories was 85.7% within the patients classified as high risk by Oncotype DX and 38.1% within the patients classified as high risk by MammaPrint (63). In another comparative study, Nunes and colleagues tested both gene signatures in 29 patients: two had high risk both by RS and MammaPrint; eight had intermediate RS, with four high risk by MammaPrint; 19 had a low RS, with eight high risk by MammaPrint. They concluded that RS and MammaPrint offer different prognostic information (64). Possible explanations for the observed variation in risk stratification include differences in baseline characteristics of the study cohorts, differences in tumor biology and/or differences in assay technology. Interestingly, among patients who undergo the Oncotype DX 21-gene assay, 39–67% receive an intermediate risk result. Recently, in the PROMIS clinical trial it was shown that 45% of intermediate risk patients have a low risk result with MammaPrint and 55% had a high risk result. The MammaPrint 70-gene signature led to change in physicians' treatment decisions in this population with early breast cancer (65).

Dowsett and colleagues compared Oncotype DX with Prosigna/PAM50 and the latter assay provided superior prognostic result on relapse risk, showing improved stratification in the intermediate and high-risk groups of patients (50). Recent preliminary results were reported for the prospective phase III clinical trial OPTIMA, which will compare Oncotype DX, MammaPrint, Prosigna, IHC4, IHC4-Aqua (NextCourse Breast), and MammaTyper gene signatures in the same group of patients (66). In this study, divergences were detected in patients attributed into risk stratification groups and molecular subtypes. The low-risk group of Oncotype DX showed a higher number of patients than the low/intermediate risk group in the Prosigna, Mammaprint or IHC4 assays and discordant molecular subtyping was observed in 40.7% of tumors. The main OPTIMA trial initiated patient enrolment in January 2017.

A deeper investigation of TransATAC study comparing the performance of the prognostic multigene signatures Oncotype DX, Prosigna/PAM50, BCI, EPClin, IHC4, and the Clinical Treatment Score showed that in patients with node negative disease the Prosigna/PAM50, BCI, and EPClin signatures provide superior prognostic accuracy, whereas BCI and EPClin provided superior prognostic accuracy for patients with node positive disease. Of note, particularly in women with node-positive disease, multigene prognostic tests when combined with clinical features significantly improved prognostic value for distant relapses and risk stratification. These results point to the importance of combining clinical and pathological information with the use of genomic signatures (13, 67).

## A multigene signature for triple negative carcinomas

Although generally seen as clinically very aggressive, triple negative breast carcinomas represent a very heterogeneous group of tumors regarding prognosis. Recently, triple negative carcinomas were stratified into molecular subtypes. Lehmann and colleagues subdivided this group of breast cancers into Basal-like (BL)-1, BL2, Mesenchymal-like (M), Mesenchymal stem-like (MSL), Luminal androgen receptor (LAR), and Imunnomodulatory (IM) (12, 30). BL-1 and IM tumors in general have a better prognosis, as they respond well to antracyclin, taxanes, and cysplatin chemotherapy and immune checkpoint activation therapy, respectively.

From the several attempts to design prognostic molecular signatures for triple negative cancers, it still remains to be clarified if obtaining a “good” prognosis group of triple negative carcinomas will make them eligible for suspending or removing the benefits of chemotherapy. Ring and colleagues developed a new classification signature based on 101 genes using Lehmann's gene expression dataset. Ring et al. (68) and a genetic assay is being developed on this algorithm building on predicting prognosis and response to therapy (69). Although distant recurrence high-risk and low-risk groups can be formulated based upon data about immune cell, inflammation response and DNA damage/repair mechanisms, the practical utility of this observation is someway restricted because even patients categorized as “good prognosis” have about 20% risk of distant recurrence in the absence of systemic adjuvant therapy (70). Of note, a higher than 10% risk of recurrence in the low risk group is too high for most patients and physicians do not support that adjuvant chemotherapy should be suspended (14). Nevertheless, developing predictors in triple negative cancers based on genetic signatures that identify patients with low risk of recurrence after completing adjuvant chemotherapy could be informative (71), allowing a better management of the disease by clinicians and patients as well. One subgroup of triple negative breast cancer patients with better outcome can be identified by administering chemotherapy before surgery. Pathologic complete response detects patients who have superior long-term survival with a given chemotherapy (72). Genetic signatures for triple negative tumors could potentially identify this disease subset and other subsets of patients with better outcome.

The utmost clinical necessity for triple negative cancers could be to develop more effective new drugs. If there are actionable molecules within certain subsets of triple negative carcinomas, this may lead to a change into a more targeted and improved therapy. Of note, it is important to refer that the triple negative carcinoma is highly heterogeneous regarding its clonal composition. Such heterogeneity explains, in part the disappointing performance of current therapies in this subtype of breast carcinomas, as resistant clones expand (69). It is possible that many triple negative carcinomas do not have an actionable mutation (73) and for these tumors there is a huge importance of obtaining both mutations profile and gene expression subtype in parallel to explore subtype specific therapies.

## Conclusion

As traditional clinical, pathological and immmunohistochemistry markers remain a standard for guiding the use of treatment, the clinician may be confronted with equivocal results that require additional testing. Such situation is epitomized in patients with luminal, HER2 negative, early stage breast carcinomas with up to 3 lymph node metastases, where the benefit of adjuvant chemotherapy is not well-defined. Gene signatures obtained through gene expression analysis, bioinformatic tools, and clinical trials can now aid the clinician in estimating the absolute benefits expected from systemic adjuvant chemotherapy or extension of adjuvant endocrine therapy. With novel and ongoing clinical trials providing a higher level of evidence of the different multigene prognostic assays, prospective decision-making based on multigene testing will pave the way for the future of treatment decision, more accurate staging of the patient and molecular subtyping of the disease. Further, molecular pathology opens new avenues for tailored therapy for each individual patient.

## Author contributions

AV and FS conceived the structure of the review and wrote the manuscript. AV performed literature screening and designed the figures and tables in consultation with FS. All authors read and approved the final manuscript.

### Conflict of interest statement

The authors declare that the research was conducted in the absence of any commercial or financial relationships that could be construed as a potential conflict of interest.

## Supplementary material

Supplementary Table 1 List of all genes/proteins present in each molecular signature. [NB, MammaPrint 70 gene signature list was obtained from Tian et al. (60)–genes in duplicate were removed, namely when different probes mapped to the same gene].

## Abbreviations

AJCC: American joint commission of cancer
ASCO: American society of clinical oncology
DFS: disease-free survival
DMFS: distant metastasis-free survival
ER: estrogen receptor
ESMO: European society of medical oncology
FDA: Food and drug administration
FFPE: Formalin-fixed paraffin embedded
HER2: Epidermal growth factor receptor 2
HR: hormone receptor
NCCN: National comprehensive cancer network
NGS: Next generation sequencing
OS: overall survival
PCR: Pathologic complete response
PgR: progesterone receptor
RFS: relapse-free survival
ROR: risk of recurrence score
RS: recurrence score
RT-PCR: reverse transcription polymerase chain reaction
TNM, tumor, node: metastasis (staging system).
